# Warts among Patients Visiting the Outpatient Department of Dermatology in a Tertiary Care Centre: A Descriptive Cross-sectional Study

**DOI:** 10.31729/jnma.7544

**Published:** 2022-09-30

**Authors:** Sunita Karki, Manish Pradhan, Anjan Rai

**Affiliations:** 1 Department of Dermatology, Nobel Medical College Teaching Hospital, Biratnagar, Morang, Nepal

**Keywords:** *genitalia*, *human papillomavirus*, *warts*

## Abstract

**Introduction:**

Warts are benign epithelial proliferations caused by a double-stranded deoxyribonucleic acid virus called human papillomavirus. They may cause significant concern and frustration on the part of the patient affecting social activities as lesions can be uncomfortable, and treatment is often painful and frustratingly ineffective. The study aimed to find out the prevalence of warts among patients visiting the outpatient Department of Dermatology in a tertiary care centre.

**Methods:**

This descriptive cross-sectional study was conducted in a tertiary care centre from 20 January 2021 to 21 February 2022. The ethical approval was taken from the Institutional Review Committee (Reference number: IRC-NMCTH 588/2021). A convenience sampling technique was used. A detailed history including distribution, size, morphology, progression and duration of the lesions was taken. Point estimate and 95% Confidence Interval were calculated.

**Results:**

Out of 4802 outpatients, 140 (2.92%) (2.44-3.40, 95% Confidence Interval) had warts. Genital wart was found in 24 (17.14%) and non-genital warts in 116 (82.85%) patients.

**Conclusions:**

The prevalence of warts among outpatients was similar when compared to other studies from similar settings.

## INTRODUCTION

Warts or verrucae are benign cutaneous and mucosal epithelial proliferations caused by papillomavirus.^1^ The incidence peak in teenage and early adult years with infection rate reaching up to 25% in some studies. Common warts may cause significant concern on the part of the patient as lesions can be uncomfortable, and treatment is often painful and frustratingly ineffective.^2^ Occupational meat handlers causing cutaneous injury, softened skin from swimming pool, nail-biting, habitual sucking of fingers, shaving and sexual activities are common modes of transmission.^3^

Even though clinical features, laboratory diagnosis and treatment are well defined for warts not much attention has been given to its prevalence and pattern in Nepal. The information extracted from studies will help us to formulate strategies to interrupt the transmission of disease and also shorten its treatment.

The objective of this study was to find out the prevalence of warts among patients visiting the outpatient Department of Dermatology in a tertiary care centre.

## METHODS

A descriptive cross-sectional study was conducted among outpatients of the Department of Dermatology of Nobel Medical College and Teaching Hospital, Biratnagar from 20 January 2021 to 21 February 2022. Ethical approval was taken from the Institutional Review Committee of the same institute (Reference number: IRC-NMCTH 588/2021). Patients visiting during the study period in the outpatient department (OPD) were included in the study. Patients unwilling to take part in the study were excluded. A convenience sampling technique was used. The sample size was calculated using the following formula:


$$\begin{array}{ll} \text{n} & ={\text{Z}}^{2}\times \frac{\text{p}\times \text{q}}{{\text{e}}^{\text{2}}}\\ & =1.96^{2}\times \frac{0.5\times 0.5}{0.02^{2}}\\ & =2401 \end{array}$$

Where,

n= minimum required sample size
Z= 1.96 at 95% Confidence Interval (CI)
p= prevalence taken as 50% for maximum sample size calculation
q= 1-p
e= margin of error, 2%

The calculated sample size was 2401. Since the convenience sampling method was done, the sample size was doubled and 4802 patients were included in the study.

The patients were diagnosed clinically to have a wart in presence of a sessile, firm, variable-sized papule, with a rough papillary surface, skin coloured or darker. A detailed history including distribution, size, morphology, progression and duration of the lesions was taken. During clinical examination, broad categorization into genital and nongenital warts and subdivision of the latter into five morphological types: common, palmoplantar, periungual, filiform and verruca plana was done.^3^ Associated family history, past history and presence of other cutaneous and systemic diseases were also noted.

Data were entered and analysed using IBM SPSS Statistics 22.0. Point estimate and 95% CI were calculated.

## RESULTS

Among 4802 outpatients, 140 (2.92%) (2.44-3.40, 95% CI) had warts. Genital wart was found in 24 (17.14%) and non-genital warts in 116 (82.85%) patients (Table 1).

**Table 1:** Morphological types of warts (n= 140).

Morphology	n (%)
Common wart	66 (47.14)
Genital wart	24 (17.14)
Palmoplantar wart	20 (14.28)
Periungual wart	9 (6.24)
Filiform wart	9 (6.24)
Verruca plana	12 (8.57)

A total of 79 (56.42%) cases of warts were present in the upper and lower limbs (Table 2).

**Table 2:** Sites of warts (n= 140).

Sites	n (%)
Genital	24 (17.14)
Nose/face	15 (10.71)
Neck/trunk	8 (5.71)
Arm/forearm/hand	29 (20.71)
Palm	10 (7.14)
Periungual	7 (5)
Thigh/leg/foot	21 (15)
Sole	12 (8.57)
Scalp	14 (10)

A total of 59 (42.14%) patients had no specific complaints while common complaints among patients found were pain 34 (24.28%), cosmesis 25 (17.86%), itching 23 (16.43%) and bleeding three (2.14%). Among patients with warts 31 (22.14%) patients presented within 3 months of onset, 25 (17.85%) presented between 3-6 months of onset, 37 (26.42%) presented between 6-12 months of onset, 42 (30%) presented between 1-3 years of onset and 5 (3.57%) presented after 3 years of onset of disease.

There were 59 (42.14%) females and 81 (57.86%) males. Overall male to female ratio was 1.37:1. The mean age was 30.11±13.94 years. Within the age group of 11-40 years, patients were 105 (75%) (Table 3).

**Table 3:** Age-wise distribution of patients with warts (n= 140).

Age group	n (%)
<10 years	5 (3.57)
11-20 years	34 (24.28)
21-30 years	44 (31.42)
31-40 years	27 (19.28)
41-50 years	13 (9.28)
>51 years	17 (12.14)

Warts was seen in 65 (46.42%) students followed by 28 (20%) servicemen/businessmen and 24 (17.14%) housewives (Table 4).

**Table 4:** Occupation of the patients with warts (n= 140).

Occupations	n (%)
Housewife	24 (17.14)
Student	65 (46.42)
Service/business	28 (20)
Worker/farmer	12 (8.57)
Retired	5 (3.57)
Health professional	3 (2.14)
Driver	3 (2.14)

## DISCUSSION

Out of 4802 patients, the prevalence of warts in OPD in the Department of Dermatology was 2.92%. A similar prevalence of warts was reported in a study conducted in Cairo, Egypt where the prevalence was 2.3%, although 2-20% prevalence has been reported in various studies.^4,5^ In our study common warts (47.14%) were the commonest type followed by genital warts (17.14%) and palmoplantar warts (14.28%). Similar findings could be seen in many other studies.^6,7^

In a study done in Pondicherry, India, extremities were involved in 57.72%, head/neck was affected in 24.9%, genitals in 6.87% and trunk in 11.36%.^8^ In a study conducted in Mangalore, India, the most common site of involvement was fingers in 44.5% followed by face/neck in 28.2% and extremities in 17.1%.^9^ These findings are almost similar to our studies where extremities were most commonly involved in 56.42% followed by genitals in 17.14%, and nose/face (10.71%). Extremities were more commonly involved due to increased chances of trauma and consequent infection in these sites.

Around 15.8% of patients reported having had clinical symptoms, including itching in 3.7%, pain during activity in 11.2%, spontaneous pain in 0.5%, and both pain during activity and spontaneous pain in 0.5% in a study done in Beijing, China.^7^ However, in our study 42.14% with no specific complaints whereas others visited us with complaints like pain (24.48%), cosmesis (17.86%), itching (16.43%) and bleeding (2.14%).

In a study done in Karnataka, India, the duration of disease was less than 3 months in 33%, 3-6 months in 20%, 6-12 months in 14% and 1-3 years in 24%.^10^ The finding was similar to our study where 22.14% of patients presented within three months of onset, 17.85% presented between 3-6 months of onset, 26.42% presented between 6-12 months of onset, 30% presented between 1-3 years of onset and 3.57% presented after 3 years of onset of disease. Although the duration of warts was variable, more than 84% of females presented within one year of onset in relation to males (53%). The late presentation of these cases in the hospital can be due to the slow and benign course and the asymptomatic nature of warts.

In a study conducted in Pune, India, the age of the patients ranged from 9-67 years where 82% of patients belonged to the 2^nd^ to 4^th^ decades.^5^ Similarly, in anotherstudy done in Karnataka, India, 75.5% of patients fell in the 11-40 years age group.^10^ Both the findings are consistent with our study where patients' age ranged from 7-64 years with 75% of patients in the 11-40 years age group. This relatively increased prevalence of warts in this age group could possibly be the result of their increased propensity to trauma facilitated-inoculation. About 55% were boys and 45% were girls in a study conducted in Asyut, Egypt, which is almost similar to our study where 57.86% were males and 42.14% were females.^6^ This can be attributed to increased outdoor activities of men. Although in another study done in Leiden, Netherland, 51% of females were affected while 49% of males were affected.^11^

Occupational stratification showed maximum being in the student group (42.7%) followed by housewives (13.7%) in a study done in Mangalore, India.^9^ In a study conducted in Pune, India, the most common occupation group was students (32%), labourers (28%) and housewives (16%).^5^ Both studies correlate well with our findings of being highest in students (46.42%) followed by servicemen/businessmen (20%) and housewives (17.14%). The higher incidence among the students could be attributable to the fact that they are physically more active and therefore at a higher risk of damage to the stratum corneum that serves as an entry point for the human papillomavirus.

The results represent the prevalence in a single tertiary centre of Eastern Nepal which may not be representative to the larger population. Studies involving a larger sample is recommended for future studies which could give a better picture of the epidemiology of warts in the community.

## CONCLUSIONS

The prevalence of warts in our study was similar when compared to other studies from similar settings. Common warts were the most common variant and students were the most common group of patients in our study.
